# RETRACTION: Etiological Characteristics of Wound Infection in Severe Trauma Patients and Logistic Regression Analysis of Influencing Factors of Infection

**DOI:** 10.1111/iwj.70630

**Published:** 2025-04-21

## Abstract

**RETRACTION:** PanX. , MoL. , and HeX. , “Etiological Characteristics of Wound Infection in Severe Trauma Patients and Logistic Regression Analysis of Influencing Factors of Infection,” International Wound Journal 21, no. 2 (2024): e14682, 10.1111/iwj.14682.PMC1083091440261155

The above article, published online on 31 January 2024, in Wiley Online Library (http://onlinelibrary.wiley.com/), has been retracted by agreement between the journal Editor in Chief, Professor Keith Harding; and John Wiley & Sons Ltd. Following an investigation by the publisher, all parties have concluded that this article was accepted solely on the basis of a compromised peer review process. In addition, further investigation by the publisher found that the article contains contradictory statements in the methodology and discussion, that many references are irrelevant and do not support the statements made in the article, and that the authors did not provide adequate ethical approval or informed consent information for the study. The editors have therefore decided to retract the article. The authors did not respond to our notice regarding the retraction.

**RETRACTION:** X. Pan , L. Mo , and X. He , “Etiological Characteristics of Wound Infection in Severe Trauma Patients and Logistic Regression Analysis of Influencing Factors of Infection,” International Wound Journal 21, no. 2 (2024): e14682, 10.1111/iwj.14682.PMC1083091440261155

The above article, published online on 31 January 2024, in Wiley Online Library (http://onlinelibrary.wiley.com/), has been retracted by agreement between the journal Editor in Chief, Professor Keith Harding; and John Wiley & Sons Ltd. Following an investigation by the publisher, all parties have concluded that this article was accepted solely on the basis of a compromised peer review process. In addition, further investigation by the publisher found that the article contains contradictory statements in the methodology and discussion, that many references are irrelevant and do not support the statements made in the article, and that the authors did not provide adequate ethical approval or informed consent information for the study. The editors have therefore decided to retract the article. The authors did not respond to our notice regarding the retraction.

